# Knockdown of GRP78 Promotes Apoptosis in Pancreatic Acinar Cells and Attenuates the Severity of Cerulein and LPS Induced Pancreatic Inflammation

**DOI:** 10.1371/journal.pone.0092389

**Published:** 2014-03-18

**Authors:** Yong Liu, Lie Yang, Ke-Ling Chen, Bin Zhou, Hui Yan, Zong-Guang Zhou, Yuan Li

**Affiliations:** 1 Department of Gastroenterological Surgery, West China Hospital, Sichuan University, Chengdu, Sichuan, China; 2 Institute of Digestive Surgery and State Key Laboratory of Biotherapy, West China Hospital, Sichuan University, Chengdu, Sichuan, China; Juntendo University School of Medicine, Japan

## Abstract

Acute pancreatitis (AP) is a potentially lethal disease characterized by inflammation and parenchymal cell death; also, the severity of AP correlates directly with necrosis and inversely with apoptosis. However, mechanisms of regulating cell death in AP remain unclear. The endoplasmic reticulum (ER) chaperone protein GRP78 has anti-apoptotic properties, in addition to modulating ER stress responses. This study used RNA interference (RNAi) approach to investigate the potential role of GRP78 in regulating apoptosis during AP. *In vitro* models of AP were successfully developed by treating AR42J cells with cerulein or cerulein plus lipoplysaccharide (LPS). There was more pancreatic inflammation and less apoptosis with the cerulein plus LPS treatment. Furthermore, knockdown of GRP78 expression markedly promoted apoptosis and reduced necrosis in pancreatic acinar cells. This was accomplished by enhancing the activation of caspases and inhibiting the activity of X-linked inhibitor of apoptosis protein (XIAP), as well as a receptor interacting protein kinase-1(RIPK1), which is a key mediator of necrosis. This attenuated the severity of pancreatic inflammation, especially after cerulein plus LPS treatment. In conclusion, these findings indicate that GRP78 plays an anti-apoptotic role in regulating the cell death response during AP. Therefore, GRP78 is a potential therapeutic target for AP.

## Introduction

Acute pancreatitis (AP) is an inflammatory disorder of the exocrine pancreas which has a range of severity and causes considerable morbidity and mortality [1]. Parenchymal cell death is a major pathologic consequence of AP. It is generally believed that mild AP is associated with extensive apoptosis, whereas severe AP is primarily associated with necrosis and relatively little apoptosis [2]–[4]. However, the mechanisms regulating cell death remain unclear.

The 78 kDa glucose regulated protein (GRP78), also referred to BiP or HSPA5, is a general chaperone that modulates unfolded proteins and endoplasmic reticulum (ER) stress responses [5]–[7]. GRP78 is also a part of the anti-apoptotic molecular cell death signaling pathway [8]–[10]. Activation of ER stress signaling concurrent with increased GRP78 was observed in an arginine induced model of severe AP [11], [12]. In contrast, reduction of GRP78 occurred in a cerulein induced in vitro model of mild AP [13]. Our previous study showed that GRP78 expression was associated with apoptosis and severity of pancreatic inflammation where in vitro models of AP induced by cerulein or cerulein plus LPS [14]. These findings indicate that the expression of GRP78 is closely related to the severity of AP. Then, question was raised that could GRP78 serve as an effective treating target for AP? Thus, RNAi based original study was designed to explore if inhibiting the expression of GRP78 could be beneficial for the treatment of AP.

In the present study, the role of GRP78 in AP was investigated using lentivirus-mediated RNA interference (RNAi) to specifically silence the expression of GRP78 in AR42J cells (rat pancreatic acinar cells). This cell line is well-characterized and widely used as in vitro models for studying secretion, apoptosis, and pancreatic inflammatory responses of the exocrine pancreas [15]. We then stimulated these cells and wild-type cells with cerulein or cerulein plus lipoplysaccharide (LPS) to induce in vitro models of AP. The secretion of digestive enzymes, pro-inflammatory cytokines levels and cell death response were analyzed.

## Materials and Methods

### Lentivirus-mediated stable RNA interference of GRP78 in AR42J cells

Rat pancreatic acinar AR42J cells (ATCC, Manassas, VA) were cultured in Ham’s F-12K medium (Hyclone) with 20% fetal bovine serum (Hyclone), L-Glutamine (0.1 mg/ml, Sigma), and antibiotics (100 U/ml penicillin and 0.1 mg/ml streptomycin) at 37°C in a humidified atmosphere containing 5% CO2 standard conditions [16]. A lentivirus carrying short hairpin RNA that targeted GRP78 gene (GenBank 14050) or that did not have an RNA interference effect (product No.SHC002, Sigma) was constructed by cotransfecting 293T cells (ATCC, Manassas, VA), with lentiviral packaging plasmids pCMV-dR8.2 dvpr (Sigma) and pCMV-VSVG (Sigma), using calcium phosphate precipitation [17], designated as LV-shGRP78 and LV-control, respectively. The short hairpin RNA sequences targeted GRP78 were the following: sense, 5′-TCAAGGTCTACGAAGGTGA-3′ and antisense, 5′- TCACCTTCGTAGACCTTGA-3′. AR42J cells were transduced with lentiviral vectors using the spinoculation method [18]. Briefly, 5.0×10^5^ cells were seeded in triplicate in six-well plates and incubated for 24 h. The medium of each well was subsequently replaced with 2 ml viral suspension in the presence of 5 μg/ml polybrene (Sigma). The multiplicity of infection of the virus was approximately 2–3.The plates were centrifuged for 1.5 h (20°C, 1200 g), followed by 5 h incubation under standard conditions and replacement with fresh medium. After 48 h of incubation, AR42J cells were passaged into 25 cm^2^ flasks and incubated under standard conditions. The enhanced green fluorescent protein (GFP) expression of transduced cells was observed using an Olympus IX81 fluorescent microscope. GFP positive cells that stably transduced with lentiviral vectors were selected using fluorescence-activated cell sorting (FACS) (BD Biosciences). The effects of knockdown on GRP78 expression were analyzed using western blot and real time reverse transcriptase PCR.

### Cell treatment and harvest

Three kinds of AR42J cell line, wild-type, LV-control and LV-shGRP78 treated cells (1×10^6^ ) were seeded in six-well plates and incubated for 24 h. Cells were then treated with 10 nmol/L cerulein (Sigma) (cerulein group) in the culture medium. Some cultures also contained 10 mg/L LPS (Sigma) (cerulein + LPS group). Phosphate buffered saline (PBS) at comparable concentrations was added to the control cultures (control group). Then, AR42J cells were cultured at various time points up to 24 h. The culture supernatant was collected to detect the secretion of amylase, lipase, and pro-inflammatory cytokines, including tumor necrosis factor-alpha (TNF-α) and interleukin 6 (IL-6) using enzyme linked immunosorbent assay (ELISA) at 4 h,8 h and 24 h after cerulein or cerulein plus LPS treatment. For cell death analysis, 1×10^5^ cells were collected to detect apoptotic and necrotic indices using flow cytometery at 24 h after cerulein or cerulein plus LPS treatment. The cell extracts were harvested at 8 h and 24 h for western blot analysis and real time RT-PCR analysis of cell death signaling molecules. These molecules included the effector caspase-3, initiator caspases-8 and -9, the endogenous caspase inhibitor X-linked inhibitor of apoptosis protein (XIAP), and receptor interacting protein kinase-1(RIPK1). Each experiment was performed in triplicate.

### Quantitative real time reverse transcriptase PCR

Total RNA was extracted from AR42J cells using TRIzol (Invitrogen), followed by reverse transcription with a DNA reverse transcription system (Invitrogen). PCR was subsequently performed in the presence of specific primers to the cDNA of rat genes. The primers were as follows: GRP78- 5′-GAAACTGCCGAGGCGTAT-3′ (F) and 5′-ATGTTCTTCTCTCCCTCTCTCTTA-3′ (R);

Amylase- 5′-AATTGATCTGGGTGGTGAGC-3′ (F) and 5′-CTTATTTGGCGCCATCGATG-3′ (R); Lipase- 5′-AGCCCAGCACAAATCAACA-3′ (F) and 5′- TCAATGAAGCCGTGGATAA-3′ (R); Caspase-3- 5′-CGGACCTGTGGACCTGAAA-3′ (F) and 5′-GGGTGCGGTAGAGTAAGC-3′ (R); Caspase-8- 5′-ACTCGGCGACAGGTTACA-3′ (F) and 5′-GGCAGCCAGTTCTTCGTT-3′ (R); Caspase-9- 5′-ACGACCTGACTGCTAAGAAA-3′ (F) and 5′-AGCCATGAGAGAGGATGAC-3′ (R); XIAP- 5′-TGTGAGTGCTCAGAAAGATAAT-3′ (F) and 5′-TGCTTCTGCACACTGTTTACA-3′ (R); RIPK1- 5′-ACCGCGCTGAGTACAATGA-3′ (F) and 5′-TCTCCACGATTATCCTTCCTT-3′ (R); β-actin -5′-CGTGAAAAGATGACCCAGAT-3′ (F) and 5′-ACCCTCATAGATGGGCACA-3′ (R). Conditions for all PCRs were optimized on an iCycler IQ (Bio-Rad) system for a 30 μl reaction as well as for the following 40-cycle program (94°C for 20 s, 53°C for 30 s, and 72°C for 30 s). β-actin was included in each reaction as an internal standard, and relative quantitative gene expression was calculated using the 2^−△△Ct^ method [19]. Each sample was analyzed in triplicate.

### Enzyme linked immunosorbent assay

The levels of amylase, lipase, TNF-α and IL-6 in the culture supernatant were determined using commercially available ELISA kits (R&D Systems) according to manufacturer’s protocols. Each sample was measured in triplicate with a microplate reader (Bio-Rad, M550).

### Western blot

Total proteins were extracted using a total protein extraction kit (KeyGen Biotech.), and protein concentrations were determined using a bicinchoninic acid protein assay kit (Pierce). Each 20 μg aliquot of total protein was loaded in a 12% sodium dodecyl sulfate-polyacrylamide gel electrophoresis gel, and then transferred onto polyvinylidene difluoride membranes (Millipore). After complete protein transfer, the membranes were blocked with 5% milk powder solution for 2 h and incubated with primary antibodies overnight. Primary antibodies used in this study included polyclonal GRP78 (Abcam), monoclonal caspase-3, XIAP, and RIPK1 (Cell Signaling) in a 1∶1000 dilution. For internal reference, a monoclonal rabbit anti-rat β-actin antibody (1∶1000 dilution) (Cell Signaling) was used. After washing the membranes, goat polyclonal anti-rabbit immunoglobulin G secondary antibody (Cell Signaling) conjugated to horseradish peroxidase was applied in a 1∶5000 dilution and incubated for 2 h at room temperature. Finally, antibody binding was visualized using the enhanced chemiluminescence system (Pierce).

### Flow cytometry

AR42J cells (1×10^5^) were collected to detect apoptotic and necrotic indices using the APC Annexin V Kit (BD Biosciences) in conjunction with the vital dye 7-amino-actinomycin (7-AAD) (BD Biosciences). Briefly, 1×10^5^ of AR42J cells were trypsinized, washed twice with clod PBS, then resuspended in 500 μl 1x binding buffer. Samples received 5 μl each of APC Annexin V and of 7-AAD, were gently vortexed, and incubated for 15 min at 25°C in the dark. Each sample was subsequently analyzed in triplicate by using flow cytometry (BD Biosciences).

### Statistical analysis

Data were expressed as means ± standard error (SE), and the differences between 3 or more groups were evaluated by one-way ANOVA. Student’s t-test was used for two group comparisons. Results were considered significant where *P*<0.05 (2-tailed).

## Results

### Effects of LV-shGRP78 mediated knockdown on GRP78 expression

**Figure 1:**
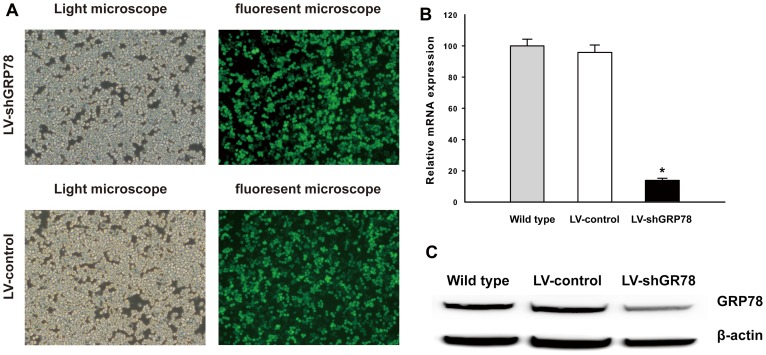
The efficacy of LV-shGRP78 mediated RNAi in AR42J cells. (A), The stable transduction efficiencies of LV-shGRP78 and LV-control in AR42J cells were detected using light microscopy and fluorescent microscopy. Bar indicates 100 μm. (B), Real time RT-PCR analysis. The expression of GRP78 mRNA was reduced by 85% in AR42J cells after LV-shGRP78 treatment, compared with wild-type cells. * p<0.05. (C), Western blot. The expression of GRP78 protein was reduced by LV-shGRP78 and was not affected by LV-control. Data shown represent at least three independent experiments.

Highly efficient transduction (> 90%) of LV-shGRP78 in AR42J cells, 48 h after infection, was documented with enhanced GFP expression observed with a fluorescent microscope (Figure 1A). Using FACS, we selected GFP positive cells that stably transduced with lentiviral vectors. The LV-control and LV-shGRP78 showed similar transduction efficiencies (Figure 1A). Quantitative real time RT-PCR documented that LV-shGRP78 reduced GRP78 mRNA expression by approximately 85% in AR42J cells, compared to wild-type cells. In contrast, the LV-control had no significant effect on GRP78 mRNA expression (Figure 1B). Western blot revealed a reduction in GRP78 protein expression in LV-shGRP78 treated cells, and no difference was evident between LV-control treated cells and wild-type cells (Figure 1C).

### Effects of GRP78 knockdown on secretion and mRNA expression of digestive enzymes

**Figure 2:**
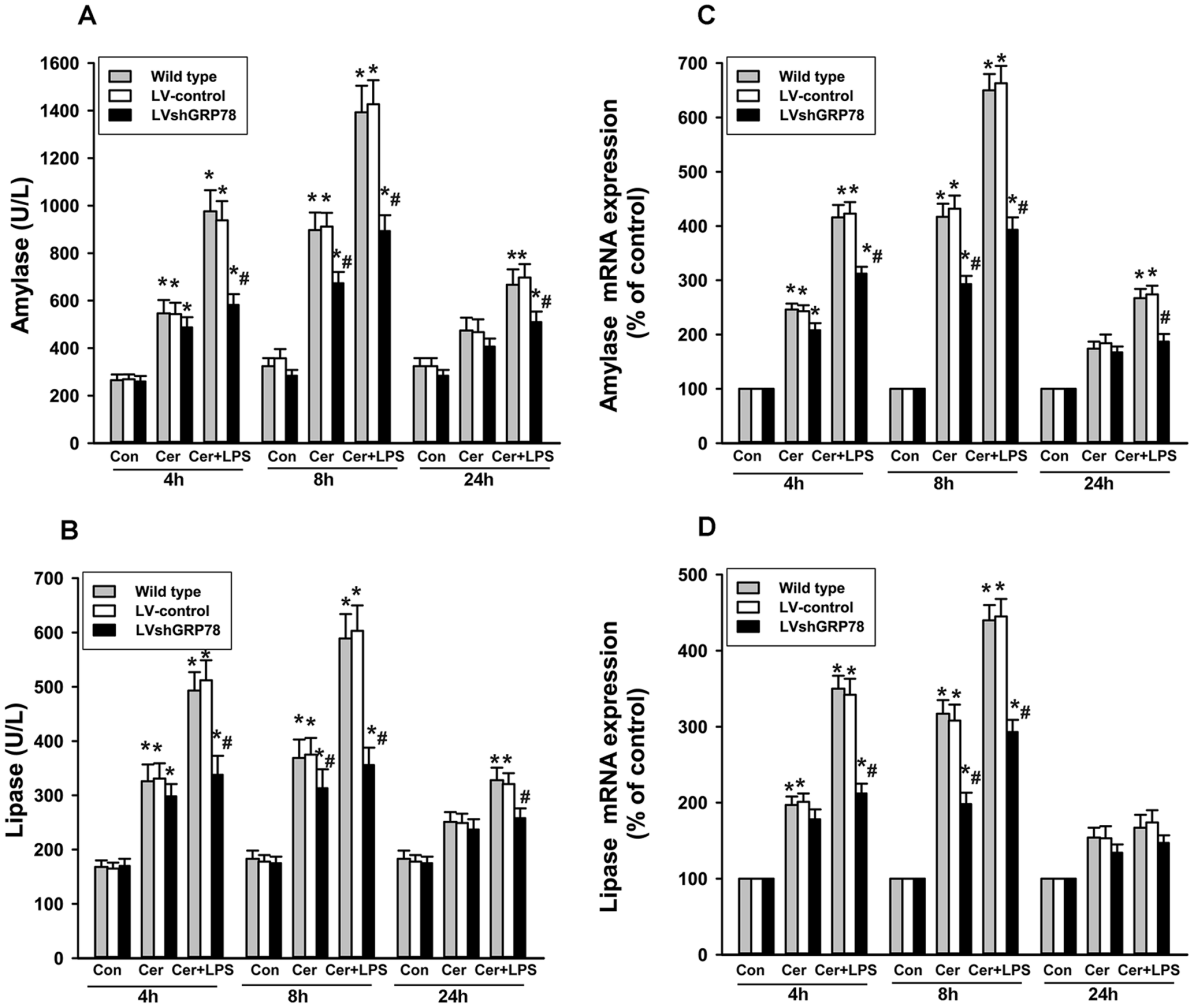
Amylase and lipase secretion levels and mRNA expressions. The levels of amylase (A) and lipase (B) in the culture supernatant were measured by ELISA at 4 h, 8h and 24 h. The mRNA expressions of amylase (C) and lipase (D)were measured by real time RT-PCR at 4h, 8 h and 24 h. Results are expressed as mean ± SE. Con: treated by PBS as control; Cer: treated by cerulein; Cer+LPS: treated by cerulein plus LPS. * p<0.05, vs. respective control; *# p*<0.05, vs. LV-control cells after the same treatment. Data shown represent at least three independent experiments.

LV-control cells treated by cerulein, with or without LPS, had enhanced amylase and lipase levels compared with controls, and this increases were more pronounced with cerulein plus LPS treatment (Figure 2AB). There were no significant differences between LV-control cells and wild-type cells. In contrast, LV-shGRP78 treated cells had a significant reduction in amylase and lipase levels, compared with LV-control cells, after cerulein plus LPS treatment, whereas only a slight difference was observed between LV-control cells and LV-shGRP78 cells treated only with cerulein. This finding was also further confirmed by real time RT-PCR analysis of amylase and lipase mRNA expression (Figure 2CD).

### Effects of GRP78 knockdown on inflammatory response

**Figure 3:**
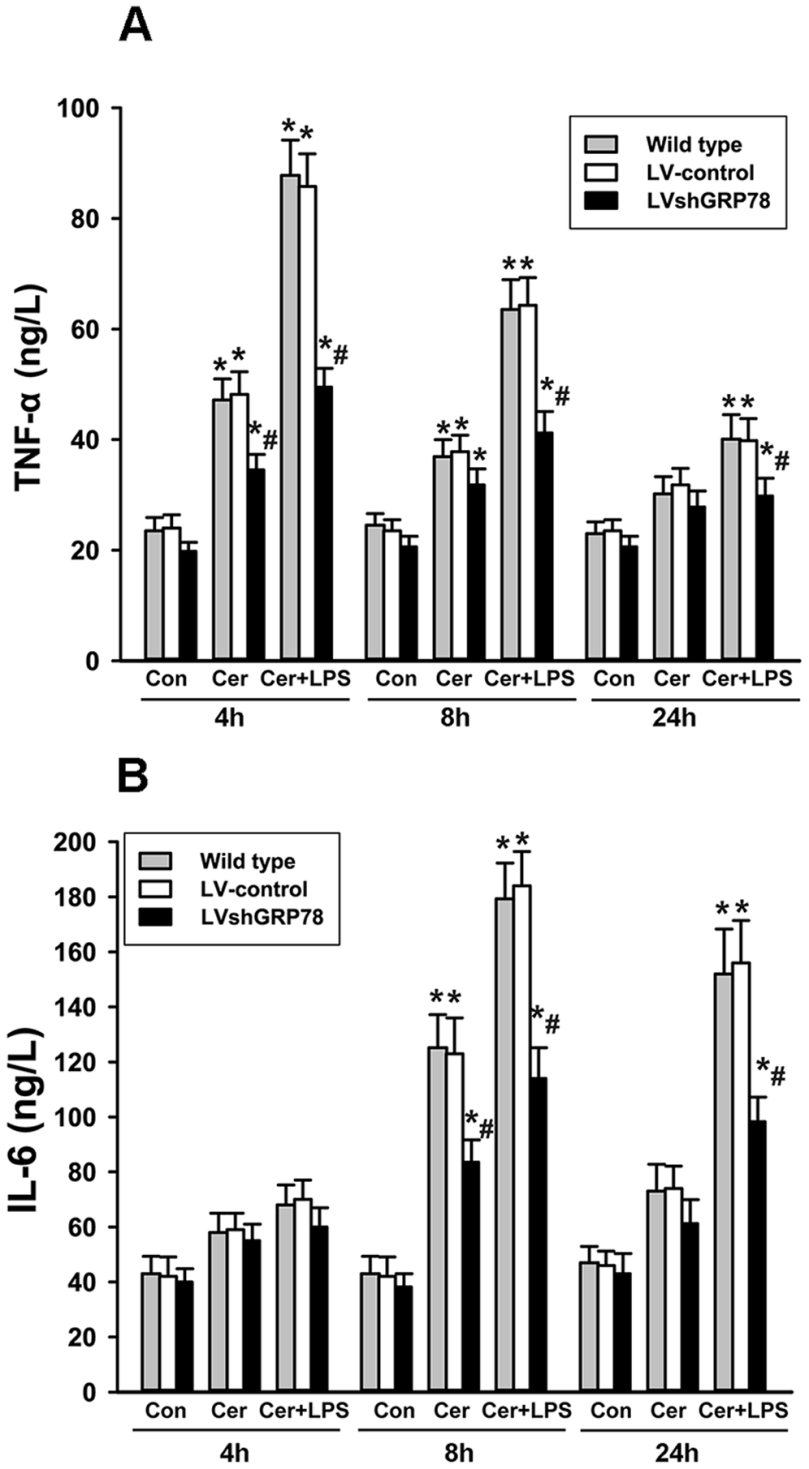
Cytokine levels in the culture supernatant after treatment measured by ELISA. (A),TNF-α levels were measured by ELISA at 4 h, 8h and24 h; (B), IL-6 levels were measured at 4h, 8 h and 24 h. Results are expressed as mean ± SE. * p<0.05, vs. respective controls; *# p*<0.05, vs. LV-control cells after the same treatment. Data shown represent at least three independent experiments.

To assess pancreatic inflammatory response, the levels of pro-inflammatory cytokines TNF-α and IL-6 were determined. TNF-α levels were markedly increases after cerulein or cerulein plus LPS treatment, compared with controls. This increase was dramatically greater after cerulein plus LPS treatment. In contrast, knockdown of GRP78 resulted in significantly reduced levels of TNF-α, compared with LV-control cells, after cerulein plus LPS treatment, and there was only a slight reduction of TNF-α levels 4 h after treatment with only cerulein (Figure 3A). LV-control cells treated with cerulein plus LPS had a pronounced increase in IL-6 levels compared with the controls. This increase was significantly reduced in the LV-shGRP78 treated cells. Treatment with cerulein alone induced a time dependent increase of IL-6 levels in LV-control cells. And the moderate increases in IL-6 levels at 8 h were reduced by GRP78 knockdown (Figure 3B).

### Effects of GRP78 knockdown on cell death

**Figure 4:**
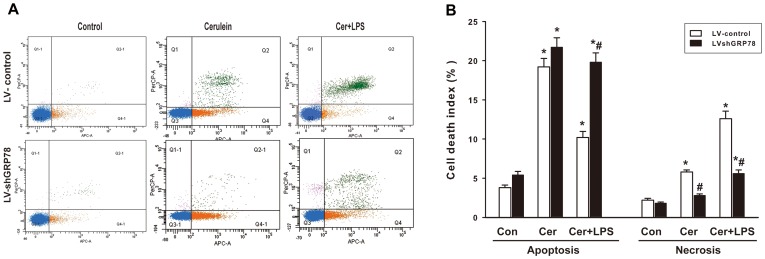
Cell death in AR42J cells after treatment was measured by flow cytometry. (A), Flow cytometric analysis of AR42J cells stained with annexin V-APC and 7-AAD at 24 h, apoptotic cells are presented in the right-lower quadrant of the figure (Q4), necrotic cells in the right-upper quadrant (Q2), living cells in the left-lower quadrant (Q3), and cell debris in the left-upper quadrant(Q1). (B), Analytical data showing the apoptotic indices and necrotic indices in both LV-control and LV-shGRP78 treated cells. Results are expressed as means ± SE. Con: treated by PBS as control; Cer: treated by cerulein; Cer+LPS: treated by cerulein plus LPS. * p<0.05, vs. the control group; *# p*<0.05, vs. LV-control cells after the same treatment. Data shown represent at least three independent experiments.

Flow cytometry of LV-control treated cells indicated that treatment with cerulein plus LPS induced a significant increase in the apoptotic index, compared with control groups, although the response was much lower than for treatment with cerulein alone (Figure 4A). This increase was significantly enhanced in LV-shGRP78 treated cells after treating with cerulein plus LPS. No significant difference was observed after LV-shGRP78 cells were treated with only cerulein, as only an increasing trend was detectable (Figure 4B). The necrotic index was markedly increased after cerulein plus LPS treatment, compared with controls. Cerulein alone induced a moderately increased necrotic index in LV-control cells. In contrast, the increase induced by cerulein or cerulein plus LPS was significantly reduced in LV-shGRP78 treated cells (Figure 4B).

### Effects of GRP78 knockdown on apoptotic signaling pathways

**Figure 5:**
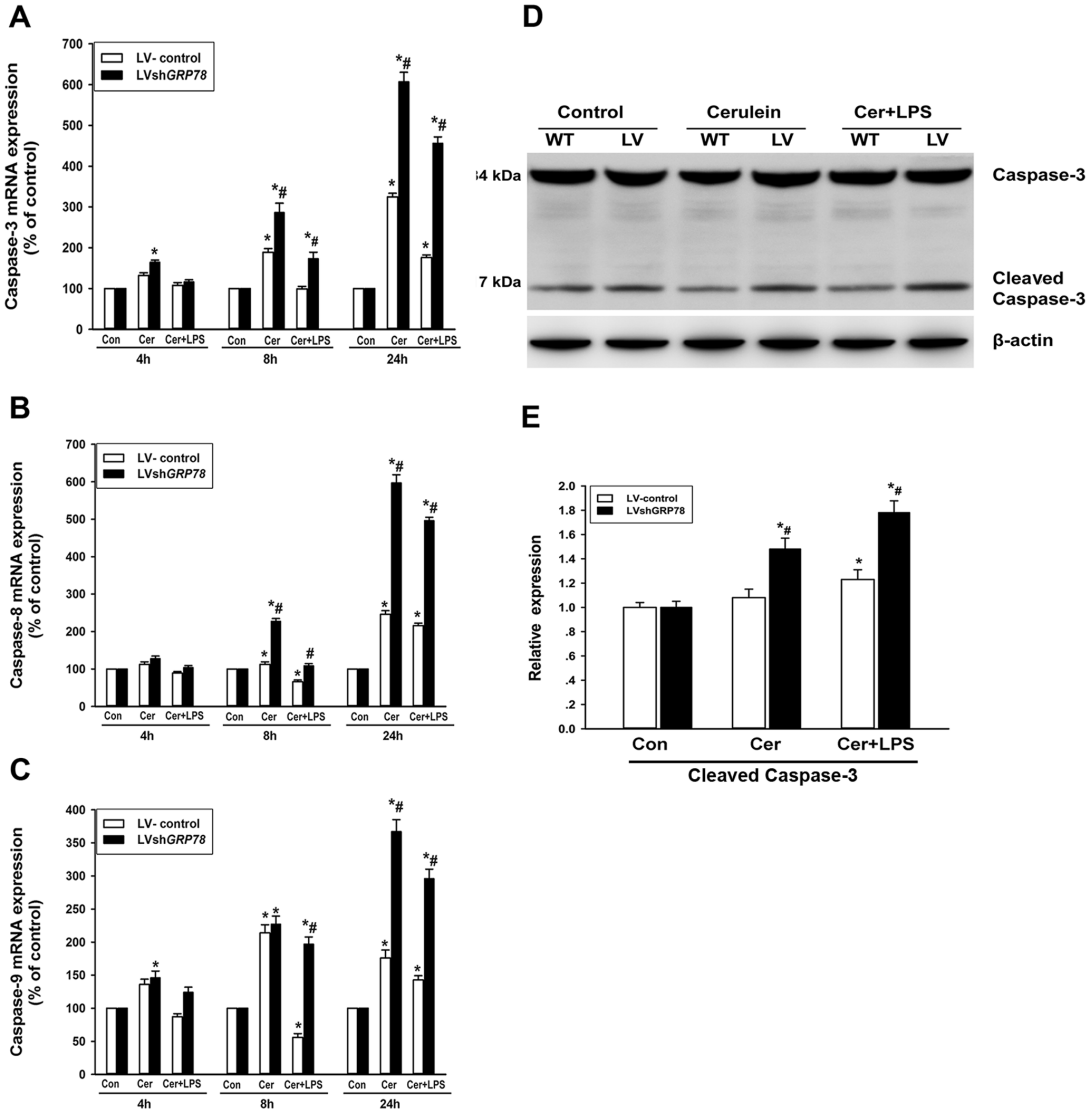
Expressions of caspases in AR42J cells after treatment. The mRNA expressions of caspases were measured by real time RT-PCR at 4h, 8 h and 24 h. Data are expressed as the percentage of the control. (A), caspase-3 mRNA levels; (B), caspase-8 mRNA levels; (C), caspase-9 mRNA levels. (D), the activity of caspase-3 at 24 h after cerulein or cerulein + LPS treatment was measured by western blot analysis. The cleaved caspase-3(17 kDa) represents the active form of caspase-3, and β-actin was used as a loading control. (E), relative quantitation data of cleaved caspase-3 protein expression. Results are expressed as means ± SE. Con: treated by PBS as control; Cer: treated by cerulein; Cer+LPS: treated by cerulein plus LPS. * p<0.05, vs. the control group; *# p*<0.05, vs. LV-control cells after the same treatment.

To assess the pro-apoptotic signaling pathways, the effector caspase-3 and initiator caspase-8 and -9 mRNA expressions as well as caspase-3 activity were determined. Caspase-3 mRNA levels in LV-control cells had time dependent increases after cerulein or cerulein plus LPS treatment, and these increases were more pronounced after treatment with only cerulein. In contrast, knockdown of GRP78 showed significantly enhanced mRNA levels of caspase-3, compared with LV-control cells, after cerulein or cerulein plus LPS treatment (Figure 5A). Furthermore, caspase-8 and -9 mRNA levels revealed a transient decrease at 8 h and a slight increase at 24 h after cerulein plus LPS treatment. Cerulein alone induced a time dependent increase of caspase-8, -9 mRNA levels in LV-control cells. Compared with LV-control cells, the mRNA levels of caspase-8 and -9 were significantly enhanced in LV-shGRP78 treated cells after cerulein or cerulein plus LPS treatment (Figure 5BC). Evidence that GRP78 knockdown promoted the activation of caspases induced by cerulein or cerulein plus LPS was confirmed by increased levels of activated caspase-3 in LV-shGRP78 treated cells, compared with LV-control cells after cerulein or cerulein plus LPS treatment (Figure 5DE).

**Figure 6:**
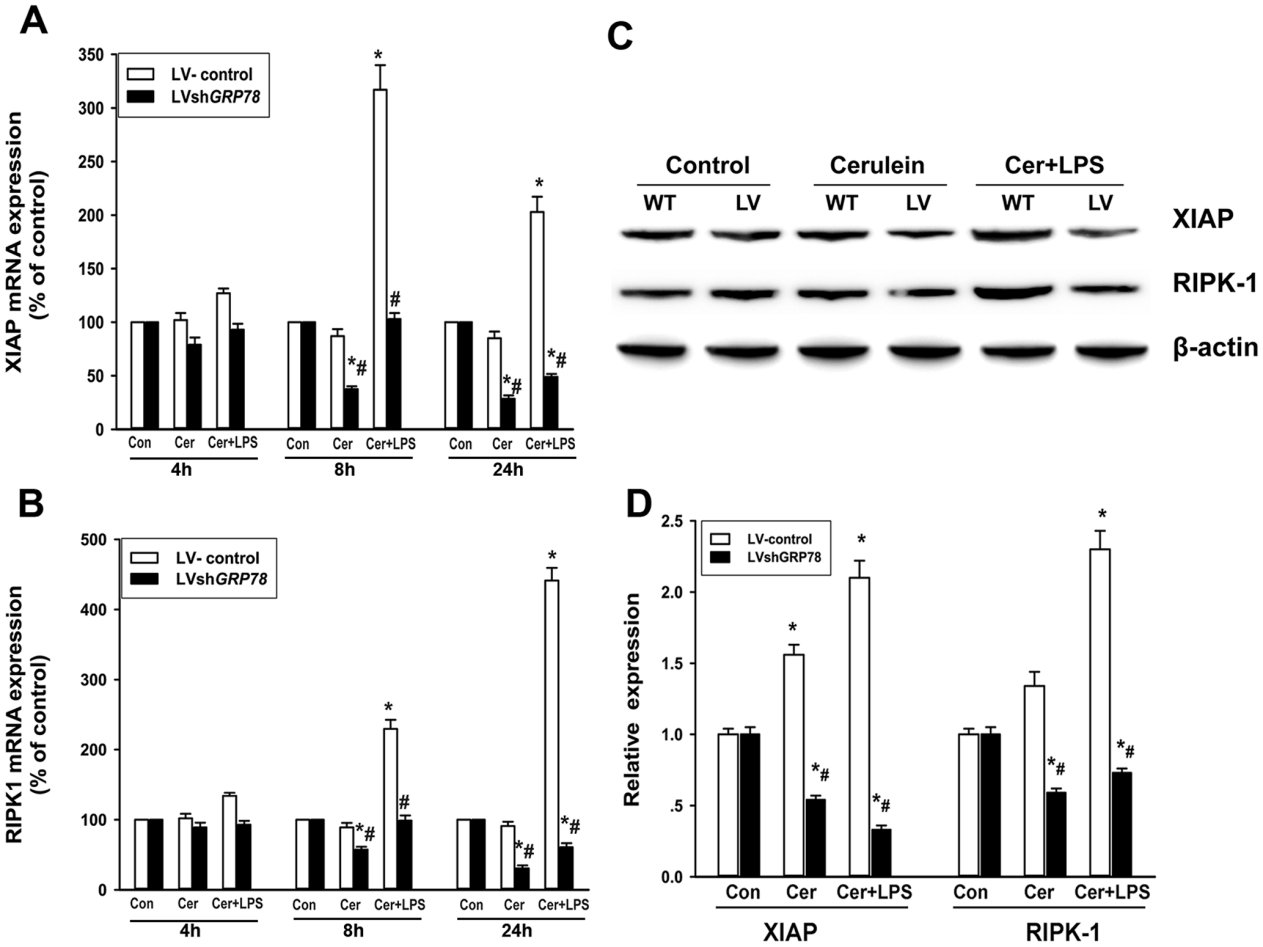
Expression of XIAP and RIPK1 in AR42J cells after treatment. The expressions of XIAP mRNA levels (A) and RIPK1 mRNA levels (B) were measured by real time RT-PCR at 8 h and 24 h. Data are expressed as the percentage of the control. (C),Western blot analysis showed the protein expressions of XIAP and RIPK1 at 24 h after cerulein or cerulein + LPS treatment with β-actin as a loading control. (D), relative quantitation data of XIAP and RIPK1 protein expression. Results are expressed as means ± SE. Con: treated by PBS as control; Cer: treated by cerulein; Cer+LPS: treated by cerulein plus LPS. * p<0.05, vs. the control group; *# p*<0.05, vs. wild-type cells after the same treatment.

Furthermore, real time RT-PCR analysis showed that XIAP mRNA levels had a time dependent increase, compared with controls, after cerulein plus LPS treatment in LV-control cells. This increase was significantly reduced in the LV-shGRP78 treated cells, with a significant decrease evident 24 h after treatment with cerulein plus LPS, compared with controls (Figure 6A). LV-control cells treated only with cerulein had no obvious changes in XIAP mRNA levels, compared with controls. In contrast, knockdown of GPR78 resulted in reduced levels of XIAP after treating only with cerulein (Figure 6A). This finding was confirmed by western blot analysis (Figure 6CD), where XIAP protein levels were significantly degraded in LV-shGRP78 treated cells, compared with LV-control cells, 24 h after cerulein or cerulein plus LPS treatment.

### Effects of GRP78 knockdown on necrotic mediators

In LV-control cells, the mRNA levels of RIPK1 had significant increases after cerulein plus LPS treatment, while no significance was observed between cells treated only with cerulein and controls. In LV-shGRP78 treated cells, the mRNA levels of RIPK1 were significantly reduced after treatment with cerulein or cerulein plus LPS, compared with LV-control cells (Figure 6B). This finding was also further confirmed by western blot analysis of RIPK1 protein expression (Figure 6CD).

## Discussion

The development of AP is a complex process that is characterized by inflammation and parenchymal cell death [20], as well as dysregulation of intracellular digestive enzyme secretion. We induced AR42J cells to mimic varying degrees of AP severity. Our results indicated that AR42J cells induced by cerulein alone had mild pancreatic inflammation with moderately elevated levels of amylase, lipase, TNF-α and IL-6, as well as high rates of apoptosis. In contrast, AR42J cells induced by a combination of cerulein and LPS had severe pancreatic inflammation with much secretion of digestive enzymes and pro-inflammatory cytokines, less apoptosis, and substantial evidence of necrosis. Furthermore, our laboratory previously demonstrated that GRP78 expression was significantly activated after cerulein plus LPS treatment, and slightly decreased after treatment with only cerulein [14]. Therefore, the model of cerulein plus LPS induced pancreatic inflammation is more useful for studying the potential anti-apoptotic role of GRP78 in AP.

We established a lentivirus-mediated RNAi system for expression of GRP78 in AR42J cells. We found that mRNA expression and protein expression of GRP78 could be silenced by the transduction of a lentivirus carrying specific short hairpin RNA. In contrast, transduction of a nontarget lentivirus had no effect on GRP78 expression in AR42J cells. Thus, we applied the convenience of lentivirus-mediated RNAi for a functional study of GRP78 regulation of cell death [21].

Apoptosis and necrosis are seen in clinical and experimental AP [3]. Apoptosis is manifested by the formation of an apoptotic body, and is reported to be non-inflammatory. In contrast, necrosis may be inflammatory, and is characterized by swelling of the cell and its organelles, as well as rupture of the plasma membrane. This results in release of cellular contents, such as digestive enzymes and inflammatory mediators [22], [23]. Therefore, necrotic death is more harmful to pancreatic acinar cells than is apoptotic death. Induction of apoptosis can relieve the harmful effects of necrosis during AP [24], [25]. In our study, knockdown of GRP78 resulted in enhanced induction of apoptosis after cerulein plus LPS stimulation. Interestingly, this knockdown also attenuated elevations in amylase and lipase that are induced by cerulein with LPS. It is believed that elevations in secretion of digestive enzymes are correlated with the degree of AP [26].

Dysregulation of digestive enzyme secretion and the inflammatory response are the initial steps in the development of AP [27]. TNF-α is a pro-inflammatory cytokine with multiple biological activities, and increasing levels are proportional to the severity of AP [28]. Furthermore, TNF-α was recently reported to play a dual role in regulating apoptosis during acute pancreatitis; a low concentration of TNF-α can induce apoptosis, whereas a high concentration causes acinar cell necrosis [29], [30]. We saw, after cerulein plus LPS treatment, that reduction of TNF-α levels induced by GRP78 knockdown was associated with an increase in apoptosis and a decrease in necrosis. IL-6 has been shown to be elevated in experimental and clinical AP [31], [32] with increased levels proportionate to increased severities of AP [33]. Chao et al. [34] indicated that blockade of IL-6 can accelerate acinar cell apoptosis and attenuate the severity of a mouse model of severe AP induced by cerulein plus LPS. Furthermore, a recent study showed that GRP78 was involved in decreased IL-6 secretion from astrocytes after lead treatment [35]. We demonstrated that levels of IL-6 were significantly reduced by GRP78 knockdown after cerulein plus LPS treatment. This suggests that GRP78 knockdown attenuates the severity of pancreatic inflammation induced by cerulein plus LPS, possibly by the promotion of pancreatic acinar cell apoptosis.

Apoptosis is a form of programmed cell death that is the consequence of several interlinked intracellular signaling pathways that include members of the caspases family [36], [37]. There are two main apoptotic pathways. The extrinsic (death receptors) and the intrinsic (mitochondrial) pathways are activated by caspase-8 and caspase-9, respectively. Activated caspase-8 and -9 subsequently cleave and activate executioner caspases, such as caspase-3 and caspase-7, which subsequently cleave intracellular substrates that cause apoptosis [38]–[40]. GRP78 has an anti-apoptotic role when it forms complexes with caspase-7 and caspase-12, thereby preventing their activation and release [8], [41]. GRP78 can also inhibit cytochrome c release from the mitochondria via interactions with anti-apoptotic Bcl-2 family members such as Bcl-2 and Bcl-X_L_ [42], [43]. Thus, we suggest that inhibition of GRP78 expression may induce apoptosis. Indeed, Fu et al. [9] indicated that reduction of GRP78 by RNAi induced apoptosis in breast cancer cells. Our study indicated that activation of caspases-3, -8, and -9, which mediate the apoptotic signaling pathway, was enhanced by GRP78 knockdown after cerulein plus LPS stimulation. Additional reports indicate that GRP78 plays an anti-apoptotic role, possibly via interactions with XIAP. XIAP is the most potent endogenous caspase inhibitor among the IAPs family, and it can inhibit mitochondria-driven caspases-3, -7, and -9 [44]–[46]. Misra et al. [47] indicated that both GRP78 and XIAP are upregulated in 1-LN prostate cancer cells, and are associated with decreased caspases activity and apoptosis. In contrast, silence of GRP78 gene expression by RNAi downregulated the expression of XIAP and upregulated apoptosis. In our study, XIAP mRNA and protein expression were significantly inhibited by GRP78 knockdown after cerulein plus LPS stimulation.

Necrosis was long regarded as an unregulated and uncontrollable process. Recent data show that necrosis may occur in a regulated manner [48]. So-called programmed necrosis (or necrosis-like programmed cell death) is mediated by death receptors such as receptor-interacting protein kinases -1 (RIPK1) and -3 (RIPK3). RIPK3 interacts with RIPK1 to form a necrosis-inducing complex that actively disintegrates mitochondrial, lysosomal, and plasma membranes, thereby resulting in necrosis [49], [50]. Moreover**,** it has been shown that levels of RIPK are directly correlated with the extent of necrosis in cerulein induced AP [4]. In this study, we demonstrated that levels of RIPK1 were significantly reduced by GRP78 knockdown after cerulein plus LPS stimulation, and were accompanied by a significant reduction in necrosis. Mareninova et al. [4] also believed that RIPK was negatively regulated by caspases, which block necrosis in cerulein induced AP. Taken together, these findings suggest that knockdown of GRP78 induces apoptosis, as opposed to necrosis, while enhancing activation of caspases and inhibiting the activity of XIAP and RIPK1 in cerulein and LPS induced AP.

In conclusion, our study provides evidence that specific knockdown of GRP78 promoted apoptosis in AR42J cells, accompanied by reduction of necrosis. This attenuates the severity of pancreatic inflammation induced by cerulein and LPS. These findings suggest that GRP78 plays an anti-apoptotic role in the regulation of the cell death response during AP, and provides a potential therapeutic target for AP.
